# The effect of nonpharmaceutical interventions on COVID-19 infections for lower and middle-income countries: A debiased LASSO approach

**DOI:** 10.1371/journal.pone.0271586

**Published:** 2022-07-22

**Authors:** Akbar Zamanzadeh, Tony Cavoli

**Affiliations:** UniSA Business School, University of South Australia, Adelaide, SA, Australia; Flinders University, AUSTRALIA

## Abstract

This paper investigates the determinants of COVID-19 infection in the first 100 days of government actions. Using a debiased LASSO estimator, we explore how different measures of government nonpharmaceutical interventions affect new infections of COVID-19 for 37 lower and middle-income countries (LMCs). We find that closing schools, stay-at-home restrictions, and contact tracing reduce the growth of new infections, as do economic support to households and the number of health care workers. Notably, we find no significant effects of business closures. Finally, infections become higher in countries with greater income inequality, higher tourist inflows, poorly educated adults, and weak governance quality. We conclude that several policy interventions reduce infection rates for poorer countries. Further, economic and institutional factors are important; thereby justifying the use, and ultimately success, of economic support to households during the initial infection period.

## 1. Introduction

The initial spread of COVID-19 infections presented a series of policy challenges for governments and public health authorities–particularly over the composition and possible magnitude of non-pharmaceutical intervention for policymakers to consider. Of all possible options, which are likely to incur economic as well as political costs? Which ones are effective? This paper investigates the determinants of COVD-19 infection rates by looking specifically at the first 100 days of government actions for addressing the spread of COVID-19 infections. In fact, analyzing the first 100 days is important to understand how governments react to control the spread of the virus when the problem has not been heightened. The first 100 days of the coronavirus challenge can be known as a "golden period" for government response because the virus has not prevailed throughout the community. Indeed, the concept of evaluating public policy during "the first 100 days" is a common term among policymakers since the US President, Franklin Roosevelt took his office in 1933. The economy faced a hard depression characterized by the high unemployment rate and bank failures when he started working as the President of the U.S. He passed 76 laws with regard to speeding up economic activities during his first 100 days of work. Performing accurate policy in the first 100 days arguably shows presidential success. Indeed, when the early actions are visible and well established, the policy will be credible for long-term plans.

We focus our study of 37 low to middle-income countries (LMCs) as these countries are characterized by high-income inequality, lack of financial resources, weak governance quality, and fragile health systems. These challenges are said to deepen the health crisis brought about by the pandemic in LMCs.

We analyze the effect of possible determinants on the spread of COVID-19 through three broad channels. First, we examine how government nonpharmaceutical interventions might impact the COVID-19 infection rate in LMCs by considering *policy actions* such as school and business closures, stay-at-home requirements, contact tracing, and testing policy. Secondly, we explore whether government *economic support* to households diminishes the infection rate in LMCs. Thirds, we examine how *socio-economic factors* such as density of population, average years of adults’ education, income inequality, international tourism arrivals, health care workers, and institutional quality affect the risk of infection. There have been many excellent contributions to this line of research examining the effects of individual variables, including income inequality (eg, [1]), governance quality [2], tourism flows [3], business closures [4], health care infrastructure [5], population density [6], government interventions [7, 8] and many others. Rather than emphasizing individual variables of interest and then focusing on issues of causality pertaining to that specific variable, we take a data-driven approach by examining many covariates to uncover the most important potential determinants of infection rate.

As such, we need to firstly deal with the issue of using many covariates in a regression analysis. We are interested in whether we can *meaningfully* reduce the number of covariates that might help determine infection rates in LMCs. As mentioned in [9–11], applying conventional regression methods like OLS with too many covariates leads to overfitting because the retained variables capture the noise of the regression. These researchers address regularizing the regression and reducing its dimension by using the least absolute shrinkage and selection operator (*LASSO*) method. This technique prevents overfitting and generates reliable out-of-sample forecasts when the model has many explanatory variables, and where non-contributing variables are eliminated from the list of potential determinants.

However, more recent studies [12–16] state that the coefficients extracted by LASSO are biased due to the correlation between covariates. To overcome this, they propose the de-biased LASSO approach. The asymptotic variance of the *de-biased LASSO* estimator is lower than that conventional *LASSO* estimator [14]. Although these researchers establish their model using time series, this paper develops the de-biased LASSO model within panel data formwork by taking account into unobserved heterogeneities across sections and over time. We employ the *de-biased LASSO* method introduced by [12] in this paper. This approach produces de-noised coefficients and allows the selection of the most influential variables explaining the variation in COVID-19 infection rates among LMCs thus providing reliable guidelines for policymaking. Moreover, we perform several robustness tests to ensure the stability of our findings.

We find that school closures, stay-at-home restrictions, and contact tracing can reduce the spread of the Coronavirus in LMCs. However, business closure is not statistically significant for lowering the COVID-19 infection rate as a nonpharmaceutical health intervention. The spread of the virus has negatively correlated with economic support to households, but this effect is not as large as the effects of other nonpharmaceutical interventions. Interestingly, a greater number of health workers leads to a decrease in the number of new infections. Further, our findings show that the more tests infer more infections. Conversely, countries with high-income inequality, a higher arrival rate of tourists, less-educated adults, and poor governance quality experience more infections. Despite using a sample pertaining to the recent infection rates for COVID-19, we feel that these results are generalizable and can be used to inform policy actions for other infectious diseases. We conclude that, for the most part, the policy interventions employed in poorer countries can reduce infection rates. Further, the economic and institutional environment is also important, thereby justifying the use, and ultimately success, of economic support to households during the initial infection period.

The paper is structured as follows: Section 2 provides a brief literature survey and some historical context. Section 3 provides the empirical model and estimation procedure. Section 4 presents the data and the results. Section 5 concludes.

## 2. Brief literature review and some history

Numerous studies show how infectious diseases like 1918 influenza, SARS, MRSA, and Avian Flu make human disasters, create economic recessions, change demographical structure, and burden socio-economic costs on firms, households, and governments. However, only 1918 influenza is comparable with COVID-19 based on the spread contagion of the virus, the medical limitation, the effect on the respiratory system, and the similarity in government interventions via closing public events, locking down societies, and requiring wearing masks (see [17]). Further, [18] indicate that 1918 influenza is the worst-case scenario for COVID-19 outcomes. Therefore, lessons from the great influenza pandemic assist policymakers in controlling current human disaster worldwide. We review public health policy during both 1918 influenza and COVID-19 to understand how the government responses affect pandemics and the expected health outcome of these interventions.

It is shown in [19] how non-pharmaceutical interventions affect the weekly excess death rate during 1918 influenza in the U.S. They gather the weekly extra death rate for 43 cities data over 24 weeks and analyze the impact of three different interventions, namely school closure, cancellation of public gatherings, and isolation (quarantine), on the outcome of the pandemic. According to their findings, non-pharmaceutical interventions significantly mitigate the consequences of the pandemic in the United States. Additionally, they state that implementing early non-pharmaceutical health policy leads to delay in reaching peak mortality. In [20], the effect of public restrictions such as school closures and social distancing on the 1918 influenza pandemic is analysed. He uses city-level U.S. data and applies the difference-in-differences (*DiD*) method to evaluate the economic and health benefits of non-pharmaceutical interventions. His medium-run findings reveal that a significant share of people saved during the 1918 pandemic lost their lives during the upcoming years. He states that the long-run social distancing probably lowers the herd immunity in society. Further, he mentions that this public health policy reduces the death rate over the short run, especially when the death rate is at its peak. Moreover, [21] analyzes the impact of three measures of public health interventions, including school closure, prohibitions on public gatherings, and isolation on the excess death rate of 1918 influenza across 45 large U.S. cities. His findings confirm the negative and significant association between non-pharmaceutical interventions and the peak of the extra death rate. He mentions that more interventions flattened the curve for mortality during the 1918 pandemic. However, this effect is weak and insignificant on overall deaths. Barro concludes that government distancing measures to the pandemic probably delay deaths in the societies rather than removing them.

The epidemiological Susceptible Infected Recovered (SIR) model is developed in [22] to multi-age groups of people above 20 years old, including "young," "the middle-aged," and the "old". They show how different government interventions in testing and tracking and group distancing impact infections, deaths, and economic loss. Their specification allows them to evaluate the trade-off between saving lives and GDP due to implementing public health interventions. Their findings reveal that keeping the adult’s death rate below 0.2 percent needs a strict economic lockdown for more than one year and a half. As a result of this intervention, the U.S. GDP shrinks almost 40 percent for one year. They argue that the government should perform a tough and long lockdown among the most vulnerable group to control infections by maximizing economic benefit. Further, less strict lockdown should be implemented for the young and middle-aged people as the low-risk groups. They conclude that decreasing the interactions between elders and other groups and increasing testing and isolating the infected ones leads to minimized economic losses and deaths.

It is shown in [23] how the spread of Coronavirus is determined via imposing economic and behavioral restrictions in New York City. He uses the fraction of COVID-19 tests yielding a positive result to measure the spread of the virus. The data covers 177 zip codes, relying daily on one. He intelligently defines new standards for business activity and stay-at-home indices by using smartphones information. The business activity index is constructed based on the number of people who visited businesses in each zip code. The stay at home index is defined by the fraction of smartphones (people) that stayed fixed at their home location during the pandemic. Further, [23] applies the fixed effects method over calendar date and the zip code of residence to capture unobserved heterogeneities over time and sections. His findings reveal that the number of visits to local businesses is positively correlated with the positivity rate of COVID-19 tests. However, the fixed location of smartphones lowers the likelihood of the positivity rate by 2 percentage points.

The effect of the business shut down on COVID-19 deaths in Italy is investigated in [4]. They gather a substantial dataset across 4,000 Italian municipalities, which covers 222 local labor markets. They define the business shutdown as the share of the workers without any essential activities—due to COVID-19— to the number of total employees. Further, some variables like the share of working-age females, the share of high school graduates, and the population density are used as other controls. Their findings reveal that business shutdown, especially in the retail trade and hospitality sectors, significantly reduce the COVID-19 death rate. Additionally, the results confirm that performing closure restrictions one week earlier could save 25 percent of the lost lives in Italy. Further, [24] analyze whether public health interventions are efficient in Europe over the first wave of the Coronavirus pandemic. The data covers 11 European countries, including Austria, Belgium, Denmark, France, Germany, Italy, Norway, Spain, Sweden, Switzerland, and the U.K. They use school closure, prohibitions of public events, social distancing, and lockdown decreed as measures of non-pharmaceutical intervention. Their findings demonstrate that economic lockdown has a large impact on reducing virus transmission in Europe.

Overall, we find that there are a number of papers that have focused on one, or a category of possible determinants of infections. In the sections that follow, we adopt a data driven approach that allows for the selection of the most influential variables that may determine COVID-19 infection rates among LMCs.

## 3. Model and methodology

We specify a reduced form panel data model with many covariates for the first 100 days of the spread of the Coronavirus as follows,

$$Y_{ij,t}=\beta X_{ij,t}+\eta W_{ij,t}+\theta Cou_{i}+\varphi Con_{j}+\lambda t+\varepsilon_{ij,t},$$ (1)

where *Y* is the ratio of the new infections per 100,000, *X* = (*X*_*1*_, *X*_*2*_,…,*X*_*n*_) includes the vector of daily government interventions such as school and business closures, stay-at-home restriction, contact tracing, households’ economic support, and tests per population. *W* = (*W*_*1*_,…,*W*_*m*_) is the vector of country-level socio-economic factors, including population density, average years of adults’ education, income inequality, international arrivals per population, and health workers per population in i^th^ country, j^th^ countinent and t^th^ period. Accordingly, *Cou*, *Con* and *t* indicate the country, continent and time fixed effects; there are several studies addressing how employing fixed/random effects for LASSO models leads to more reliable estimates due to capturing unobserved heterogeneities [25, 26].

These fixed effects are utilized to flexibly account for omitted variables within regions and over time. The reason we employ the continent fixed effects is to capture regions’ unobserved heterogeneity, though we do run regressions without regional fixed effects as part of testing for robustness. We normalize the variables to avoid problems with scaling. [27] suggest normalization of variables because the scale of variables affect regulation of the parameters. This transformation changes the distribution of variables to a normal one with zero mean and unit variance. It means if the model consists of some categorical variables, they will no longer be discontinuous. This normalization allows us to interpret their coefficients like continuous regressors rather than interpreting each category with regard to a baseline group, case, or condition. Further, due to the normalization of variables, the model does not include an intercept. Note that these fixed effects do not lead to an unidentifiability issue in the model because the time-invariant variables such as education, income inequality, etc., vary from country to country. Therefore, the time invariants and country or continent fixed effects do not overlap each other. The parameters *β*, *η*, *θ*, *φ* and *λ* are the vectors of slopes. *ε* is the error term.

To show the procedure of *de-biased LASSO*, we first simplify our representations and specify the Eq (1) as,

Y=Zψ+ε. (2)

Where *Z* and *ψ* reflect for a p-dimensional vector of explanatory variables and their corresponding coefficients, respectively. Based on *Zhang and Zhang* (*2014*), firstly, we need to de-correlate the vector of normalized explanatory variables, *Z*. For this purpose, we employ *LASSO* fit of an explanatory variable, *Z*_*j*_ versus all other explanatory variables, *Z*_−*j*_. Here, the dimension of *Z*_−*j*_ is *p*−1. Accordingly, we set the optimization problem as follows,

$${\hat{\gamma }}_{j}=\begin{array}{c} argmin\\ \gamma \in {\mathbb{R}}^{p-1} \end{array}(\frac{∥Z_{j}-Z_{-j}\gamma {∥}_{2}^{2}}{NT})+\zeta_{Z}∥\gamma {∥}_{1}.$$ (3)

Eq (3) satisfies the Karush–Kuhn–Tucker (KKT) optimality condition for the estimates. *γ* is a vector of coefficients corresponding to *Z*_−*j*_. *ζ*>0 is a tuning parameter that is determined by a cross-validation technique. ∥.∥_1_ and ∥.∥_2_ are ℓ_1_ and ℓ_2_ norms, respectively. ℓ_1_ and ℓ_1_ norms are known as “Absolute-value” and “Euclidean” norms, as well. Generally, for a vector of *x* = (*x*_1_,⋯,*x*_*i*_), one can define ℓ_p_ norm as, ${∥x∥}_{p}={\left(\sum_{i=1}^{n}{|x_{i}|}^{p}\right)}^{1/p}$, where *p*≥1. *N* and *T* indicate the number of sections (countries)—and time. The residuals of Eq (3) are denoted by,

$$M_{j}=Z_{j}-Z_{-j}{\hat{\gamma }}_{j}.$$ (4)

Now, we regress the vector of residuals defined by Eq (4) over the response variable in Eq (2) as follows,

$$\psi_{j}^{†}=\frac{M_{j}^{Tr}Y}{M_{j}^{Tr}Z_{j}}=\psi_{j}+\underset{k\neq j}{\sum }\frac{M_{j}^{Tr}Z_{k}}{M_{j}^{Tr}Z_{j}}\psi_{k}+\frac{M_{j}^{Tr}\varepsilon }{M_{j}^{Tr}Z_{j}}.$$ (5)

Eq (5) demonstrates *M*_*j*_ is not orthogonal to *Z*_*j*_. This issue induces bias in $\psi_{j}^{†}$. The second part of the decomposition, $\sum_{k\neq j}\frac{M_{j}^{Tr}Z_{k}}{M_{j}^{Tr}Z_{j}}\psi_{k}$ refers to the bias arising from the existing correlation between the explanatory variables; it is worth noting here that the correlations among the explanatory variables in models with too many covariates lead to biased estimates using alternative regularization and variable selection methods, such as Ridge regression and elastic net [28, 29]. Here, $M_{j}^{Tr}Z_{k}\neq 0$ at least for one *k*≠*j*. The last term, $\frac{M_{j}^{Tr}\varepsilon }{M_{j}^{Tr}Z_{j}}$ is equal to zero because *M*_*j*_ and *ε* are orthogonal to each other.

In the second step, we regress Z over Y trough employing the LASSO method. The KKT optimality condition is presented below,

$${\hat{\psi }}^{initial}=\begin{array}{c} argmin\\ \psi \in {\mathbb{R}}^{p} \end{array}(\frac{∥Y-Z\psi {∥}_{2}^{2}}{NT})+\zeta_{Z}∥\psi {∥}_{1}.$$ (6)

Now, we deduct the bias term obtained Eq (5) from each component of the estimated coefficients, ${\hat{\psi }}^{initial}$. Therefore, the de-biased LASSO estimator is shown as follows,

$${\hat{\psi }}_{j}^{de-biased}={\hat{\psi }}^{initial}-\sum_{k\neq j}\frac{M_{j}^{Tr}Z_{k}}{M_{j}^{Tr}Z_{j}}\;{\hat{\psi }}_{k}^{initial},$$ (7)

where the final bias term is calculated via inserting the coefficient ${\hat{\psi }}_{k}^{initial}$ obtained from Eq (6).

We have a specific value for each coefficient after the debiasing procedure because the debiased coefficients now consist of two parts. Then, even the ${\hat{\psi }}^{initial}$ is zero, it is likely the second part of Eq (7), $\sum_{k\neq j}\frac{M_{j}^{Tr}Z_{k}}{M_{j}^{Tr}Z_{j}}\;{\hat{\psi }}_{k}^{initial}$ will not be zero.

A framework to estimate regression variance for the *debiased LASSO* method is developed in [12]. They show $\tau_{j}^{-1}({\hat{\psi }}_{j}-\psi_{j})\approx N(0,\sigma^{2})$, where $\tau_{j}=\frac{{∥M_{j}∥}_{2}}{|M_{j}^{Tr}Z_{j}|}$ is known as a "noise factor." It is shown in [13] that this condition is satisfied even for mis-specified models. Indeed, such inference allows researchers to calculate reliable confidence intervals and p-values for estimates.

## 4. Data, estimation, and results

Our dataset covers 37 LMCs across four geographic regions, including 11 African, 12 Asian, 8 Latin American and the Caribbean, and 6 European countries (See Appendix A in the S1 Appendix). The definitions and sources of the data, including measures of interventions and nonpharmaceutical instruments, government support, socio-economic conditions, and health care variables are all reported in Appendix B in S1 Appendix and a scatterplot is presented in Appendix F in S1 Appendix. We employ a low dimension debiased LASSO method (see [12]) since the number of explanatory variables—12 variables without considering the continent and country fixed effects—is less than the number of observations, 3700 = 37 countries x 100 days.

This paper considers governance quality as one of the major predictors of covid infection rate. The intuition for using such a variable is to understand how good or bad governance contributes to the control of the infection rate. As one of the limitations of this research, we did not access updated information for this variable. Accordingly, we create the governance quality index based on [30] by combining six governance dimensions via *principal component analysis* (PCA). In terms of policy analysis, this unique index is much more informative for policymaking because it reflects the rank of low and middle-income countries in governance quality. As such, countries can ascertain how their performance in governing societies contributes to combat with new diseases. Details of data and method are given in Appendix C in S1 Appendix. We also provide a table of descriptive statistics of all variables including the governance quality indicator, and a correlation matrix for the key variables in Appendix D in S1 Appendix.

We summarize the results in Table 1 where we estimate 9 different models. Model 1 is the baseline regression in which government nonpharmaceutical interventions explain the infection rate. Model 2 includes government supports to households. We evaluate the effects of other factors one by one, Model 3 to 8, to show the robustness of estimates when controls change. Model 9 presents the effects of all policy variables and socio-economic covariates.

**Table 1:** The effects of government interventions on COVID-19 infection rate.

	Model 1	Model 2	Model 3	Model 4	Model 5	Model 6	Model 7	Model 8	Model 9
**School closure**	**-0.0563** ^**^	**-0.0422** ^*^	**-0.0608** ^***^	**-0.0511** ^**^	**-0.0270**	**-0.0413** ^*^	**-0.0395** ^*^	**-0.0534** ^*^	**-0.0541** ^**^
	(0.0226)	(0.0227)	(0.0226)	(0.0230)	(0.0230)	(0.0226)	(0.0226)	(0.0229)	(0.0227)
**Business closure**	**0.0027**	**0.0010**	**-0.0038**	**0.0104**	**-0.0089**	**-0.0002**	**0.0022**	**-0.0020**	**-0.0158**
	(0.0225)	(0.0223)	(0.0220)	(0.0226)	(0.0225)	(0.0223)	(0.0222)	(0.0223)	(0.0227)
**Stay at home requirement**	**-0.1077** ^***^	**-0.0875** ^***^	**-0.0681** ^***^	**-0.0914** ^***^	**-0.0802** ^***^	**-0.0875** ^***^	**-0.0767** ^***^	**-0.0971** ^***^	**-0.0353**
	(0.0238)	(0.0242)	(0.0240)	(0.0242)	(0.0243)	(0.0241)	(0.0247)	(0.0243)	(0.0251)
**Contact tracing**	**-0.1347** ^***^	**-0.1436** ^***^	**-0.1273** ^***^	**-0.1354** ^***^	**-0.1771** ^***^	**-0.1486** ^***^	**-0.1445** ^***^	**-0.1053** ^***^	**-0.1008** ^***^
	(0.0254)	(0.0253)	(0.0251)	(0.0255)	(0.0263)	(0.0254)	(0.0252)	(0.0273)	(0.0275)
**Test per population**	**0.0036** ^***^	**0.0036** ^***^	**0.0039** ^***^	**0.0037** ^***^	**0.0037** ^***^	**0.0036** ^***^	**0.0036** ^***^	**0.0036** ^***^	**0.0039** ^***^
	(0.0001)	(0.0001)	(0.0001)	(0.0001)	(0.0001)	(0.0001)	(0.0001)	(0.0001)	(0.0001)
**Economic support to households**		**-0.0029** ^***^	**-0.0034** ^***^	**-0.0029** ^***^	**-0.0030** ^***^	**-0.0031** ^***^	**-0.0033** ^***^	**-0.0021** ^***^	**-0.0031** ^***^
		(0.0007)	(0.0007)	(0.0007)	(0.0007)	(0.0007)	(0.0008)	(0.0008)	(0.0008)
**Health care workers**			**-0.0455** ^***^						**-0.0348** ^***^
			(0.0063)						(0.0092)
**Average years of adults’ education**				**-0.0274** ^***^					**-0.0321** ^**^
				(0.0103)					(0.0168)
**Income inequality**					**0.0140** ^***^				**0.0269** ^***^
					(0.0028)				(0.0038)
**Density of population**						**0.0002** ^**^			**0.0002** ^*^
						(0.0001)			(0.0001)
**Tourism arrivals**							**0.1263** ^**^		**0.6147** ^***^
							(0.0664)		(0.0791)
**Governance quality**								**-0.1470** ^***^	**-0.3669** ^***^
								(0.0395)	(0.0484)
**Africa**	**-0.3056** ^***^	**-0.2584** ^***^	**-0.2213** ^***^	**-0.2865** ^***^	**-0.2749** ^***^	**-0.2431** ^***^	**-0.2261** ^***^	**-0.2584** ^***^	**-0.3039** ^***^
	(0.0377)	(0.0374)	(0.0375)	(0.0400)	(0.0375)	(0.0373)	(0.0372)	(0.0374)	(0.0395)
**Asia**	**-0.1717** ^***^	**-0.1340** ^***^	**-0.0767** ^***^	**-0.1340** ^***^	**-0.1471** ^***^	**-0.1221** ^***^	**-0.1112** ^***^	**-0.1340** ^***^	**-0.0695** ^***^
	(0.0368)	(0.0365)	(0.0360)	(0.0365)	(0.0366)	(0.0364)	(0.0363)	(0.0365)	(0.0357)
**Latin America**	**0.2136** ^***^	**0.2355** ^***^	**0.2880** ^***^	**0.2355** ^***^	**0.2159** ^***^	**0.2467** ^***^	**0.2515** ^***^	**0.2355** ^***^	**0.2276** ^***^
	(0.0418)	(0.0415)	(0.0409)	(0.0415)	(0.0416)	(0.0413)	(0.0412)	(0.0415)	(0.0406)
**Europe**	**0.3798** ^***^	**0.3878** ^***^	**0.5324** ^***^	**0.4460** ^***^	**0.4222** ^***^	**0.3884** ^***^	**0.3497** ^***^	**0.3878** ^***^	**0.5170** ^***^
	(0.0468)	(0.0464)	(0.0514)	(0.0508)	(0.0473)	(0.0463)	(0.0477)	(0.0464)	(0.0533)
**R** ^**2**^	**0.033**	**0.066**	**0.069**	**0.069**	**0.059**	**0.062**	**0.073**	**0.067**	**0.093**
**F-stat.**	**335.36**	**109.54**	**123.66**	**115.17**	**122.81**	**10167**	**106.89**	**95.21**	**168.23**
**No. Obs.**	**3700**	**3700**	**3700**	**3700**	**3700**	**3700**	**3700**	**3700**	**3700**

Notes: the values between parenthesizes represent the standard errors. The up scripts "***", "**", "*" reflect statistical significance for each coefficient at 99, 95 and 90 percent of credible intervals, respectively.

Our finding strongly supports that the school closure and contact tracing significantly mitigate the infection rate in LMCs. Further, the results confirm that the testing and infection rates are positively correlated with each other. More interestingly, we find that the economic support package to households is negatively associated with the infection rate. However, and notably, there is no evidence of a significant correlation between business closures and infection rates–calling into question the efficacy of that policy action for LMCs.

Since variables are standardized, coefficients must be interpreted based on the changes in standard deviations (see [31]). In Model 1 a one standard deviation hike in the school closure index–keeping other variables fixed—leads to a 0.056 standard deviation decrease in the infection rate. Also, a positive change of one standard deviation in the contact tracing policy leads to a 0.135 standard deviation decrease in the infection rate. Further, the stay-at-home restriction reduces infections by a 0.108 standard deviation. Regarding testing policy, one standard deviation hike in the testing rate by keeping other variables fixed increases the infection rate by 0.4 percent standard deviation.

Model 2 presents the coefficients for government supports to households. The incidence of infection decreases by a 0.3 standard deviation when household economic support increases by one standard deviation while keeping all other variables unchanged. From Model 3, for the effect of health care workers, increasing one standard deviation in the number of nurses and doctors decreases the infection rate by a 0.045 standard deviation. This finding is consistent with the work of [5] and [32], which shows that the efficacy of the health care system can contribute to reducing cases.

Model 4 shows that average years of adults’ schooling years have negatively correlated with the infection rate, as per [33], which examines the relationship between schooling and health-related behaviours; an "education gradient". Accordingly, we expect the virus to be less prevalent in more educated countries. If we hold other variables constant, a one standard deviation increase in adults’ average schooling years results in a 0.027 standard deviations decrease in infection rates.

Model 5 shows the effect of income inequality on the spread of the virus. Here, the nexus between income inequality and infection rate is positive–consistent with [1] and [34]. People living in countries with extreme income inequality are not able to afford primary health care. Keeping other factors constant, the infection rate increases by a 0.014 standard deviation when income inequality increases by one standard deviation.

Model 6 reports the reaction of the infection rate to population density. The infection rate increases by 0.02 percent standard deviation when the density of the population changes by one standard deviation, consistent with [6]. The magnitude of the coefficient is not considerable, although it is statistically significant. This finding may be due to a significant proportion of the economy living in rural areas characterized by a low density of people.

Model 7 demonstrates the effect of tourism arrivals on the COVID-19 infection rate. The standardized coefficient of this variable reveals that one standard deviation increase in tourism arrivals hikes the infection rates by 0.126 standard deviations. A very similar result is found in [3]. The size of this coefficient is larger than other socio-economic covariates, suggesting that controlling tourism arrivals may have allowed governments to prevent the spread of the virus before implementing a national policy a border closures. It is worth pointing out here that border closures were not implemented until well into the first 100 days of COVID cases. As such, there was still a significant flow of tourists during that time. See https://www.bsg.ox.ac.uk/research/research-projects/covid-19-government-response-tracker, and https://www.bbc.com/news/world-52103747.

Model 8 shows the importance of the quality of public institutions in controlling COVID-19 outbreaks. The estimated coefficient reveals that the infection rate falls by a 0.147 standard deviation when governance quality rises by one standard deviation, keeping other variables fixed. This result is consistent with [35] and [2], highlighting the role of good governance.

Model 9 provides broad estimates of coefficients when all policy variables and socio-economic controls are included in the model. The results for this regression are consistent with the above results.

### Robustness checks

We refit the model with other alternative *debiased LASSO* methods such as *scaled debiased LASSO and residual bootstrapping debiased LASSO* methods to check the consistency of the estimates. Both of these approaches are derived from Eq (6) after a few adjustments. The former modifies the penalty function to estimate the regression’s noise along with the slopes. The latter resamples the residuals obtained from Eq (6), then approximates the empirical distribution of the outcome variable. This provides more accurate confidence intervals for the estimates [36]. It is stated in [37] that using the bootstrapping technique leads to a precise selection procedure as well.

The Scaled debiased LASSO is constructed based on [38–40] to consider variance and coefficient in the optimization procedure. In light of this, the *scaled LASSO* technique jointly estimates the regression coefficients and noise level in a linear model as $\frac{\sigma }{2}+\frac{{∥Y-Z\psi ∥}_{2}^{2}}{\left(2\sigma NT\right)}+\zeta_{0}{∥\psi ∥}_{1}$. According to this scale-invariance analysis, the penalty level is proportional to the noise level of the regression model. Recall, the standard LASSO method assumes that the optimal penalty parameter depends on the error scale, so it is mainly determined by cross-validation. Therefore, the Scaled LASSO offers the advantage of scale-free penalty parameters that are predetermined from purely theoretical considerations (*see* [41]). The Scaled LASSO provides the advantage of automatically adjusting the penalty level in a regression model for yielding optimal convergence, (see [42]). Although this approach uses an alternative optimization function, it needs to be debiased like the conventional LASSO model in [12]. It is shown in [41] that the Scaled LASSO method performs inappropriately when predictors are strongly correlated with each other. The results of the Scaled debiased LASSO are provided in Table 2 as follows,

**Table 2:** Robustness test based on the scaled debiased LASSO.

	Model 1	Model 2	Model 3	Model 4	Model 5	Model 6	Model 7	Model 8	Model 9
**School closure**	**-0.0559** ^***^	**-0.0415** ^**^	**-0.0607** ^***^	**-0.0508** ^**^	**-0.0266**	**-0.0408** ^*^	**-0.0389** ^*^	**-0.0530** ^***^	**-0.0543** ^***^
	(0.0219)	(0.0222)	(0.0223)	(0.0225)	(0.0223)	(0.0222)	(0.0222)	(0.0223)	(0.0224)
**Business closure**	**0.0025**	**0.0007**	**-0.0043**	**0.0102**	**-0.0095**	**-0.0005**	**0.0018**	**-0.0024**	**-0.0157**
	(0.0218)	(0.0218)	(0.0217)	(0.0221)	(0.0219)	(0.0218)	(0.0218)	(0.0218)	(0.0223)
**Stay at home requirement**	**-0.1080** ^***^	**-0.0871** ^***^	**-0.0676** ^***^	**-0.0909** ^***^	**-0.0796** ^***^	**-0.0870** ^***^	**-0.0764** ^***^	**-0.0969** ^***^	**-0.0352**
	(0.0231)	(0.0236)	(0.0237)	(0.0237)	(0.0236)	(0.0236)	(0.0242)	(0.0237)	(0.0247)
**Contact tracing**	**-0.1347** ^***^	**-0.1435** ^***^	**-0.1272** ^***^	**-0.1354** ^***^	**-0.1773** ^***^	**-0.1486** ^***^	**-0.1446** ^***^	**-0.1051** ^***^	**-0.1007** ^***^
	(0.0247)	(0.0247)	(0.0247)	(0.0249)	(0.0255)	(0.0249)	(0.0247)	(0.0267)	(0.0271)
**Test per population**	**0.0036** ^***^	**0.0036** ^***^	**0.0039** ^***^	**0.0037** ^***^	**0.0037** ^***^	**0.0036** ^***^	**0.0036** ^***^	**0.0036** ^***^	**0.0039** ^***^
	(0.0001)	(0.0001)	(0.0001)	(0.0001)	(0.0001)	(0.0001)	(0.0001)	(0.0001)	(0.0001)
**Economic support to households**		**-0.0029** ^***^	**-0.0034** ^***^	**-0.0029** ^***^	**-0.0030** ^***^	**-0.0031** ^***^	**-0.0033** ^***^	**-0.0021** ^***^	**-0.0031** ^***^
		(0.0007)	(0.0007)	(0.0007)	(0.0007)	(0.0007)	(0.0007)	(0.0008)	(0.0008)
**Health care workers**			**-0.0456** ^***^						**-0.0347** ^***^
			(0.0062)						(0.0091)
**Average years of adults’ education**				**-0.0275** ^***^					**-0.0326** ^**^
				(0.0101)					(0.0166)
**Income inequality**					**0.0140** ^***^				**0.0269** ^***^
					(0.0027)				(0.0038)
**Density of population**						**0.0002** ^*^			**0.0002** ^*^
						(0.0001)			(0.0001)
**Tourism arrivals**							**0.1264** ^*^		**0.6158** ^***^
							(0.0652)		(0.0779)
**Governance quality**								**-0.1473** ^***^	**-0.3670** ^***^
								(0.0385)	(0.0477)
**Africa**	**-0.1250** ^***^	**-0.1246** ^***^	**-0.1744** ^***^	**-0.1584** ^***^	**-0.1811** ^***^	**-0.1257** ^***^	**-0.1219** ^***^	**-0.1475** ^***^	**-0.3362** ^***^
	(0.0366)	(0.0365)	(0.0370)	(0.0391)	(0.0365)	(0.0366)	(0.0365)	(0.0365)	(0.0389)
**Asia**	**-0.0357**	**-0.0365**	**-0.0371**	**-0.0373**	**-0.0372**	**-0.0373**	**-0.0372**	**-0.0372**	**-0.0398**
	(0.0357)	(0.0356)	(0.0355)	(0.0357)	(0.0356)	(0.0357)	(0.0356)	(0.0356)	(0.0351)
**Latin America**	**0.3216** ^***^	**0.3314** ^***^	**0.3664** ^***^	**0.3309** ^***^	**0.2550** ^***^	**0.3309** ^***^	**0.3243** ^***^	**0.3328** ^***^	**0.2116** ^***^
	(0.0406)	(0.0405)	(0.0403)	(0.0405)	(0.0405)	(0.0405)	(0.0405)	(0.0405)	(0.0400)
**Europe**	**0.3743** ^***^	**0.3848** ^***^	**0.5846** ^***^	**0.4382** ^***^	**0.4227** ^***^	**0.3847** ^***^	**0.3496** ^***^	**0.3888** ^***^	**0.5434** ^***^
	(0.0455)	(0.0454)	(0.0507)	(0.0497)	(0.0460)	(0.0454)	(0.0468)	(0.0453)	(0.0525)
**R** ^**2**^	**0.019**	**0.053**	**0.068**	**0.057**	**0.053**	**0.050**	**0.063**	**0.055**	**0.097**
**F-stat.**	**335.36**	**90.01**	**133.07**	**92.24**	**101.44**	**86.03**	**92.75**	**82.11**	**172.09**
**No. Obs.**	**3700**	**3700**	**3700**	**3700**	**3700**	**3700**	**3700**	**3700**	**3700**

Notes: the values between parenthesizes represent the standard errors. The up scripts "***", "**", "*" reflect statistical significance for each coefficient at 99, 95 and 90 percent of credible intervals, respectively.

Also, for another robustness check, a residual bootstrapping method for the debiased LASSO regression is implemented. As given in [43], this process starts with estimating coefficients from the conventional LASSO method. Then, initial residuals are calculated through $\hat{\varepsilon }=Y-Z\hat{\psi }$. Accordingly, centered residuals are computed by ${\hat{\varepsilon }}_{it}^{centered}={\hat{\varepsilon }}_{it}-\bar{\hat{\varepsilon }}$ for (*i* = 1,⋯,*N*) and (*t* = 1,⋯,*T*). Also, an expected value for residuals equals $\bar{\hat{\varepsilon }}={NT}^{-1/2}\sum_{t=1}^{T}\sum_{n=1}^{N}{\hat{\varepsilon }}_{it}$. Note that the expected value for residuals is not zero in the original LASSO because the type of penalty applied for regularizing the parameters differs from the OLS.

Then, the bootstrapped errors $\left(\varepsilon_{11}^{*},\cdots ,\varepsilon_{NT}^{*}\right)$, are obtained from centered residuals. After that, the bootstrapped response variable is produced as follows,

$$Y^{*}=Z\hat{\psi }+\varepsilon^{*}.$$ (8)

Here, Y* is non-random and the sample has a fixed design now. [43] show that the estimates obtained from the residual bootstrapping debiased LASSO method are asymptotically consistent. Also, [44] indicates that the residual bootstrapping debiased LASSO estimates are more efficient than other debiased methods. The estimates for the residual bootstrapping debiased LASSO method after 500 times replications are represented below (Table 3),

**Table 3:** Robustness test by applying the bootstrapping debiased LASSO.

	Model 1	Model 2	Model 3	Model 4	Model 5	Model 6	Model 7	Model 8	Model 9
**School closure**	**-0.0563** ^**^	**-0.0423** ^*^	**-0.0609** ^***^	**-0.0511** ^**^	**-0.0270**	**-0.0415** ^*^	**-0.0394** ^*^	**-0.0533** ^***^	**-0.0540** ^**^
	(0.0226)	(0.0228)	(0.0228)	(0.0232)	(0.0229)	(0.0228)	(0.0225)	(0.0228)	(0.0228)
**Business closure**	**0.0027**	**0.0011**	**-0.0036**	**0.0104**	**-0.0089**	**-0.0002**	**0.0021**	**-0.0020**	**-0.0158**
	(0.0225)	(0.0224)	(0.0223)	(0.0228)	(0.0224)	(0.0224)	(0.0221)	(0.0222)	(0.0227)
**Stay at home requirement**	**-0.1077** ^***^	**-0.0876** ^***^	**-0.0683** ^***^	**-0.0914** ^***^	**-0.0802** ^***^	**-0.0875** ^***^	**-0.0767** ^***^	**-0.0971** ^***^	**-0.0353**
	(0.0238)	(0.0243)	(0.0243)	(0.0244)	(0.0242)	(0.0243)	(0.0245)	(0.0242)	(0.0251)
**Contact tracing**	**-0.1347** ^***^	**-0.1436** ^***^	**-0.1275** ^***^	**-0.1355** ^***^	**-0.1771** ^***^	**-0.1485** ^***^	**-0.1443** ^***^	**-0.1053** ^***^	**-0.1008** ^***^
	(0.0254)	(0.0254)	(0.0253)	(0.0257)	(0.0262)	(0.0255)	(0.0250)	(0.0272)	(0.0276)
**Test per population**	**0.0036** ^***^	**0.0036** ^***^	**0.0039** ^***^	**0.0037** ^***^	**0.0037** ^***^	**0.0036** ^***^	**0.0036** ^***^	**0.0036** ^***^	**0.0039** ^***^
	(0.0001)	(0.0001)	(0.0001)	(0.0001)	(0.0001)	(0.0001)	(0.0001)	(0.0001)	(0.0001)
**Economic support to households**		**-0.0029** ^***^	**-0.0034** ^***^	**-0.0029** ^***^	**-0.0030** ^***^	**-0.0031** ^***^	**-0.0033** ^***^	**-0.0021** ^***^	**-0.0031** ^***^
		(0.0007)	(0.0007)	(0.0007)	(0.0007)	(0.0008)	(0.0008)	(0.0008)	(0.0008)
**Health care workers**			**-0.0455** ^***^						**-0.0348** ^***^
			(0.0063)						(0.0092)
**Average years of adults’ education**				**-0.0273** ^**^					**-0.0319** ^**^
				(0.0104)					(0.0169)
**Income inequality**					**0.0140** ^***^				**0.0269** ^***^
					(0.0028)				(0.0038)
**Density of population**						**0.0002** ^*^			**0.0002** ^**^
						(0.0001)			(0.0001)
**Tourism arrivals**							**0.1260** ^**^		**0.6145** ^***^
							(0.0660)		(0.0793)
**Governance quality**								**-0.1471** ^***^	**-0.3668** ^***^
								(0.0393)	(0.0485)
**Africa**	**-0.3056** ^***^	**-0.2915** ^***^	**-0.2821** ^***^	**-0.3315** ^***^	**-0.2584** ^***^	**-0.2749** ^***^	**-0.2003**	**-0.2292**	**-0.2952** ^***^
	(0.0377)	(0.0376)	(0.0379)	(0.0403)	(0.0374)	(0.0375)	(0.0370)	(0.0372)	(0.0396)
**Asia**	**-0.1717** ^***^	**-0.1614** ^***^	**-0.1221** ^***^	**-0.1717** ^***^	**-0.1340** ^***^	**-0.1471** ^***^	**-0.0923** ^***^	**-0.1112** ^***^	**-0.0763** ^***^
	(0.0368)	(0.0367)	(0.0364)	(0.0368)	(0.0365)	(0.0366)	(0.0361)	(0.0363)	(0.0358)
**Latin America**	**0.2136** ^***^	**0.2183** ^***^	**0.2467** ^***^	**0.2136** ^***^	**0.2268** ^***^	**0.2247** ^***^	**0.2687** ^***^	**0.2571** ^***^	**0.2368** ^***^
	(0.0418)	(0.0417)	(0.0413)	(0.0418)	(0.0415)	(0.0416)	(0.0410)	(0.0412)	(0.0407)
**Europe**	**0.3798** ^***^	**0.3813** ^***^	**0.5404** ^***^	**0.4398** ^***^	**0.4233** ^***^	**0.3862** ^***^	**0.3497** ^***^	**0.3887** ^***^	**0.5204** ^***^
	(0.0468)	(0.0467)	(0.0520)	(0.0513)	(0.0471)	(0.0466)	(0.0475)	(0.0462)	(0.0534)
**R** ^**2**^	**0.033**	**0.067**	**0.074**	**0.071**	**0.058**	**0.065**	**0.071**	**0.064**	**0.093**
**F-stat.**	**335.36**	**114.65**	**136.29**	**123.11**	**120.27**	**105.82**	**102.79**	**91.29**	**168.32**
**No. Obs.**	**3700**	**3700**	**3700**	**3700**	**3700**	**3700**	**3700**	**3700**	**3700**

Notes: the values between parenthesizes represent the standard errors. The up scripts "***", "**", "*" reflect statistical significance for each coefficient at 99, 95 and 90 percent of credible intervals, respectively.

As seen, all coefficients obtained from the residual bootstrapping debiased LASSO method are in line with the estimates obtained from the debiased LASSO method. The important point about the debiased LASSO methods here is the results are close to each other. However, the initial estimates from the conventional LASSO, scaled LASSO and bootstrapping LASSO are somehow different. Appendix E in S1 Appendix reports the initial estimates for model 9 based on these different approaches.

The findings show that the estimates of the conventional and bootstrapping LASSO models are similar to each other in terms of variable selection with slight differences in the magnitude of coefficients. The results with the other method, the Scaled LASSO, differ from the conventional and bootstrapping LASSO techniques. The results reveal that the Scaled LASSO selects more variables compared to the other two methods. Additionally, the magnitude of coefficients selected by the Scaled LASSO method is slightly larger than the other two methods. These differences are due to the different penalty functions employed for selecting covariates.

Further, we check the robustness of the models by changing the sample size and implementing extreme bounds analysis (EBA). Based on [45], we first change the sample size through the *leave-one-out method* and re-estimate the models to ensure the estimates are robust and are not sensitive to the sample size. For example, we remove the first country, i.e., Argentina, from our sample and re-estimate the coefficients throughout Model 1 to 9. Then, we proceed estimating procedure by removing the second country and replacing the first country instead. This leave-one-out approach is continued to remove the last country, namely, Zimbabwe, and refit all models. Hence, we estimate 37 (samples with leaving a country out) x 9 (models) = 333 more regressions through the de-biased LASSO method. To perform EBA, we calculate the average value of each coefficient, $Avg({\hat{\psi }}_{j}^{de-biased})$ and the corresponding standard error $Avg({\hat{\sigma }}_{j}^{de-biased})$ relying on 37 different estimated regressions for each model. Then, we construct the extreme bounds for 95 percent of the credible interval via $Avg({\hat{\psi }}_{j}^{de-biased})\mp 1.96\times Avg({\hat{\sigma }}_{j}^{de-biased})$. In which, $Avg({\hat{\psi }}_{j}^{de-biased})-1.96\times Avg({\hat{\sigma }}_{j}^{de-biased})$ and $Avg({\hat{\psi }}_{j}^{de-biased})+1.96\times Avg({\hat{\sigma }}_{j}^{de-biased})$ are the lower and upper extreme bounds, respectively. This approach outlined here is similar to [46] because the extreme bounds are constructed based on the average values of coefficients obtained from different design matrices. He considers different combinations of variables and attains the average values for each coefficient. Then, establish the lower and upper extreme bounds for estimates. Likewise, we calculate these bounds relying on the average value of coefficients by altering the sample size. In this sense, a coefficient is fragile when the lower outer bound is negative while the upper extreme bound is positive. Table 4 reports the average coefficient values and the corresponding lower and upper bounds.

**Table 4:** Robustness test based on the extreme bounds analysis.

** **	**Model 1**			**Model 2**			**Model 3**			**Model 4**			**Model 5**		
** **	**Coefficient**	**Lower extreme bound**	**Upper extreme bound**	**Coefficient**	**Lower extreme bound**	**Upper extreme bound**	**Coefficient**	**Lower extreme bound**	**Upper extreme bound**	**Coefficient**	**Lower extreme bound**	**Upper extreme bound**	**Coefficient**	**Lower extreme bound**	**Upper extreme bound**
**School closure**	-0.0613	-0.0801	-0.0426	-0.0413	-0.0604	-0.0221	-0.0597	-0.0790	-0.0404	-0.0498	-0.0695	-0.0301	-0.0263	-0.0457	-0.0069
**Business closure**	0.0076	-0.0111	0.0262	0.0017	-0.0169	0.0203	-0.0033	-0.0218	0.0151	0.0105	-0.0086	0.0296	-0.0082	-0.0268	0.0104
**Stay at home requirement**	-0.1070	-0.1278	-0.0861	-0.0866	-0.1084	-0.0648	-0.0673	-0.0892	-0.0454	-0.0898	-0.1117	-0.0679	-0.0789	-0.1007	-0.0571
**Contact tracing**	-0.1273	-0.1511	-0.1035	-0.1428	-0.1667	-0.1189	-0.1266	-0.1505	-0.1027	-0.1350	-0.1592	-0.1107	-0.1762	-0.2016	-0.1508
**Test per population**	0.0036338	0.0036335	0.0036341	0.0036174	0.0036171	0.0036177	0.0038609	0.0038606	0.0038612	0.0036757	0.0036754	0.0036760	0.0036898	0.0036895	0.0036901
**Economic support to households**				-0.00289	-0.00291	-0.00287	-0.00336	-0.00338	-0.00334	-0.00286	-0.00288	-0.00284	-0.00297	-0.00299	-0.00294
**Health care workers**							-0.0448	-0.0463	-0.0433						
**Average years of adults’ education**										-0.0263	-0.0303	-0.0263			
**Income inequality**													0.01395	0.01367	0.01395
**Density of population**															
**Tourism arrivals**															
**Governance quality**															
**AFRICA**	-0.2270	-0.2793	-0.1747	-0.2614	-0.3134	-0.2094	-0.2768	-0.3301	-0.2236	-0.2873	-0.3468	-0.2277	-0.2510	-0.3027	-0.1993
**ASIA**	-0.1095	-0.1592	-0.0598	-0.1322	-0.1816	-0.0828	-0.1156	-0.1646	-0.0666	-0.1307	-0.1801	-0.0813	-0.1219	-0.1710	-0.0727
**AMERICA**	0.2562	0.1918	0.3206	0.2380	0.1740	0.3020	0.2512	0.1878	0.3147	0.2368	0.1729	0.3008	0.2326	0.1687	0.2965
**EUROPE**	0.3867	0.3059	0.4674	0.3828	0.3025	0.4630	0.5306	0.4297	0.6315	0.4395	0.3433	0.5357	0.4196	0.3373	0.5019
** **	**Model 6**			**Model 7**			**Model 8**			**Model 9**		
** **	**Coefficient**	**Lower extreme bound**	**Upper extreme bound**	**Coefficient**	**Lower extreme bound**	**Upper extreme bound**	**Coefficient**	**Lower extreme bound**	**Upper extreme bound**	**Coefficient**	**Lower extreme bound**	**Upper extreme bound**
**School closure**	-0.0407	-0.0599	-0.0215	-0.0384	-0.0576	-0.0191	-0.0525	-0.0719	-0.0330	-0.0524	-0.0717	-0.0331
**Business closure**	0.0006	-0.0181	0.0192	0.0028	-0.0158	0.0214	-0.0013	-0.0199	0.0172	-0.0154	-0.0346	0.0038
**Stay at home requirement**	-0.0861	-0.1079	-0.0642	-0.0757	-0.0987	-0.0527	-0.0960	-0.1181	-0.0740	-0.0333	-0.0568	-0.0098
**Contact tracing**	-0.1467	-0.1709	-0.1224	-0.1435	-0.1675	-0.1196	-0.1053	-0.1331	-0.0776	-0.0995	-0.1279	-0.0711
**Test per population**	0.0036383	0.0036380	0.0036386	0.0035853	0.0035851	0.0035856	0.0036287	0.0036285	0.0036290	0.0039079	0.0039076	0.0039082
**Economic support to households**	-0.00305	-0.00307	-0.00303	-0.00321	-0.00323	-0.00319	-0.00208	-0.00211	-0.00206	-0.00306	-0.00309	-0.00304
**Health care workers**										-0.0345	-0.0378	-0.0313
**Average years of adults’ education**										-0.0308	-0.0415	-0.0201
**Income inequality**										0.02654	0.02598	0.0271
**Density of population**	0.0001446	0.0001442	0.0001449							0.0001530	0.0001526	0.0001534
**Tourism arrivals**				0.1263	-0.0409	0.2934				0.6077	0.3719	0.8435
**Governance quality**							-0.1448	-0.2030	-0.0866	-0.3612	-0.4496	-0.2728
**AFRICA**	-0.2642	-0.3163	-0.2122	-0.2669	-0.3190	-0.2148	-0.2565	-0.3084	-0.2047	-0.2989	-0.3570	-0.2407
**ASIA**	-0.1323	-0.1818	-0.0828	-0.1377	-0.1872	-0.0881	-0.1276	-0.1769	-0.0783	-0.0733	-0.1207	-0.0260
**AMERICA**	0.2371	0.1730	0.3012	0.2285	0.1643	0.2926	0.2405	0.1767	0.3043	0.2311	0.1695	0.2926
**EUROPE**	0.3861	0.3057	0.4665	0.3459	0.2600	0.4318	0.3841	0.3040	0.4642	0.5104	0.4036	0.6172

According to the magnitudes and signs of the coefficients, the values in Table 2 are broadly similar to those in Table 1; all estimates related to the government interventions except the business closure are robust. This is consistent over different models. It has also been demonstrated that other socio-economic factors associated with the spread of the virus are robust across all models, as none of the structured credible intervals contain a zero value.

## 5. Conclusions

We examine the effects of several government nonpharmaceutical interventions, as well as the socio-economic environment on the spread of the virus in LMCs. We take a data-driven and multivariate approach in order to identify the important factors determining infection rates.

In order to deal with a dataset with too many predictors involving correlation between covariates, we apply a debiased LASSO method along with several robustness exercises. Our findings suggest that school closure and contact tracing are the most effective interventions compared to other government responses to the spread of COVID-19. Curiously, we found that a policy involving business closures to be statistically insignificant in affecting infection rates. Further, our results reveal that economic support to households and the number of healthcare workers negatively affect the spread of the virus.

Finally, the density of population, income inequality, and tourism arrivals contribute to infections in those countries. In contrast, average years of adults’ education and governance quality impact the infection rate negatively.

Closing schools and universities, limiting people to stay at home, and tracing the contacts of infected individuals are all effective policy interventions. Further,‌ strengthening public institutions and increasing the number of health care workers are vital to assisting these countries in overcoming this health crisis.

While this paper has employed a sample pertaining to infection rates for the recent COVID-19 pandemic, we feel that these results can be generalized to inform policy actions for other infectious diseases. We conclude that, for the most part, there are a range of policy interventions can reduce infection rates for poorer countries. Further, socio-economic factors, the economic and institutional environment are also important in impacting the spread of infections. We feel that, as a consequence, this justifies the use, and ultimately the success–as shown in our analysis, of economic support to households during the initial infection period.

## Supporting information

S1 Appendix A. List of low and middle-income countries and the date of the first incidence of COVID-19 infection. B. Definitions and sources of variables. C. Principal component analysis. Index for the governance quality. D. Descriptive statistics of variables. E. the initial estimates with the conventional, scaled, and bootstrapping LASSO methods. F. Scatter plots between infection rate and different instruments of government health policy in LMCs.
(DOCX)
