# Supplementary material for: The effect of nonpharmaceutical interventions on COVID-19 infections for lower and middle-income countries: A debiased LASSO approach
Source: PLoS One. 2022 Jul 22;17(7):e0271586. doi: 10.1371/journal.pone.0271586 (PMC9307185; doi:10.1371/journal.pone.0271586)
Supplement: S1 Appendix — A. List of low and middle-income countries and the date of the first incidence of COVID-19 infection. B. Definitions and sources of variables. C. Principal component analysis. Index for the governance quality. D. Descriptive statistics of variables. E. the initial estimates with the conventional, scaled, and bootstrapping LASSO methods. F. Scatter plots between infection rate and different instruments of government health policy in LMCs. (DOCX) [file pone.0271586.s001.docx]

**Appendix**

**Appendix A. List of low and middle-income countries and the date of the first incidence of COVID-19 infection**

| **Africa** | | **Asia** | | **Latin America & Caribbeans** | | **Europe** | |  |
| --- | --- | --- | --- | --- | --- | --- | --- | --- |
|  |  |  |  |  |  |  |  |  |
| **Country** | **Date of the first infection** | **Country** | **Date of the first infection** | **Country** | **Date of the first infection** | **Country** | **Date of the first infection** |  |
| **Cote d'Ivoire** | 12/03/2020 | **Bangladesh** | 9/03/2020 | **Argentina** | 4/03/2020 | **Belarus** | 28/02/2020 |  |
| **Ethiopia** | 14/03/2020 | **India** | 30/01/2020 | **Bolivia** | 12/03/2020 | **Bulgaria** | 8/03/2020 |  |
| **Kenya** | 14/03/2020 | **Indonesia** | 2/03/2020 | **Colombia** | 7/03/2020 | **Romania** | 27/02/2020 |  |
| **Morocco** | 3/03/2020 | **Iran** | 20/02/2020 | **Costa Rica** | 7/03/2020 | **Russia** | 1/02/2020 |  |
| **Nigeria** | 28/02/2020 | **Iraq** | 25/02/2020 | **Cuba** | 12/03/2020 | **Serbia** | 7/03/2020 |  |
| **Rwanda** | 15/03/2020 | **Kazakhstan** | 15/03/2020 | **El Salvador** | 19/03/2020 | **Ukraine** | 4/03/2020 |  |
| **Senegal** | 3/03/2020 | **Malaysia** | 25/01/2020 | **Mexico** | 29/02/2020 |  |  |  |
| **South Africa** | 6/03/2020 | **Myanmar** | 24/03/2020 | **Paraguay** | 8/03/2020 |  |  |  |
| **Togo** | 15/03/2020 | **Pakistan** | 27/02/2020 |  |  |  |  |  |
| **Tunisia** | 3/03/2020 | **Philippines** | 30/01/2020 |  |  |  |  |  |
| **Zimbabwe** | 21/03/2020 | **Thailand** | 13/01/2020 |  |  |  |  |  |
|  |  | **Turkey** | 12/03/2020 |  |  |  |  |  |

**Appendix B. Definitions and sources of variables**

| **Variable Name** | **Definition** | **Source** |
| --- | --- | --- |
| Infection rate | the ratio of covid-19 new infections per 100000 population | *Our World in Data* |
| Average years of adults' schooling | the average schooling years for adults over 25 years old | *World Bank* |
| Income Inequality | the Gini coefficient | *World Bank* |
| Density of population | the number of people per square kilometer | *World Bank* |
| Governance Quality | It is the combination of six dimensions of governance quality including, control of corruption, the rule of law, government effectiveness, rule quality, political stability, and voice and accountability. The weight of each factor is calculated via principal component analysis (PCA). | *World Bank* |
| Health Care Workers | the number of doctors and nurses per 1000 people | *World Health Organization (WHO)* |
| Tourism arrivals per population | the proportion of foreign tourism arrivals to1000 people | *World Bank* |
| School closure | It is ordinal variable recording schools and universities closures. It defines as,  0 when a government does not recommend closing  1 when a government requires closing (only some levels or categories, e.g., just high school, or just public schools)  2 when a government requires closing all levels | *Oxford COVID-19 Government Response Tracker* (OxCGRT) |
| Business closure | It is an ordinal measure of closing of workplaces. It defines as,  0 when a government does not recommend closing  1 when a government recommends closing (or work from home)  2 when a government require closing (or work from home) for some sectors or categories of workers  3 when a government requires closing (or work from home) all-but-essential workplaces (e.g., grocery stores, doctors) | *Oxford COVID-19 Government Response Tracker* (OxCGRT) |
| Stay at home requirements | It is an ordinal measure confining people to their homes. It defines as,  0 when there is no intervention.  1 when a government recommends people not to leave their houses.  2 when a government requires people not to leave their houses with exceptions for daily exercise, grocery shopping, and 'essential' trips.  3 when a government requires people not to leave their houses with minimal exceptions (e.g., allowed to leave only once a week, or only one person can leave at a time, etc.) | *Oxford COVID-19 Government Response Tracker* (OxCGRT) |
| Economic support to households | This index is a combination of 2 measures including, A) income support for households: 0 - no income support 1 - the government is replacing less than 50% of lost salary (or if a flat sum, it is less than 50% median salary) 2 - the government is returning 50% or more of lost pay (or if a flat sum, it is greater than 50% median salary) B) debt or contract relief for households: 0 - No 1 - Narrow relief, specific to one kind of contract 2 - broad debt/contract relief | *Oxford COVID-19 Government Response Tracker* (OxCGRT) |
| Contact tracing | It is an Ordinal measure of government contact tracing. It defines as,  0 when there is no contact tracing  1 when there is limited contact tracing - not done for all cases  2 when there is comprehensive contact tracing - done for all identified cases | *Oxford COVID-19 Government Response Tracker* (OxCGRT) |
| Testing rate | the ratio of the daily COVID-19 tests per 100000 population | *Our World in Data* |
|  | | |

**Appendix C. Principal component analysis. Index for the governance quality**

Principal Components Analysis (PCA) is a method for combining several variables to create a composite index. As a multivariate statistical method, PCA uses mixtures of variables to establish latent variables known as Principal Components (PA) from initial variables. Practically, PCA constructs uncorrelated components obtained from weighted linear combinations of the original variables. Assume a vector of variables, $X=(X_{1}, \cdots, X_{n)}$, hence, m principal components can be defined as follows,

$${PC}_{1}= \alpha_{11}X_{1}+\alpha_{12}X_{2}+\cdots+\alpha_{1n}X_{n}$$

$\vdots$ (1)

${PC}_{m}= \alpha_{m1}X_{1}+\alpha_{m2}X_{2}+\cdots+\alpha_{mn}X_{n}$ ,

in which $\left( \alpha_{11}, \cdots, \alpha_{ij} \right)$, *i=(1, …, n)* and *j=(I, …, m)*, are the corresponding weights to the i^th^ variable into the j^th^ principal component. Weights can be obtained by calculating the eigenvalues of the correlation matrix or the covariance matrix if the original variables are standardized. Eq. (1) satisfies three conditions as,

1. All principal components, $({PC}_{1}, \cdots, {PC}_{m})$, are orthogonal to each other.
2. The first principal component captures the greatest proportion of the variance of the variables. Accordingly, the second component accounts for most of the remaining variance. This will continue until the final principal component absorbs all of the difference not covered by the preceding component.
3. The sum of all squared weights equals one, i.e., $\alpha_{11}^{2}+\alpha_{12}^{2}+ \cdots+\alpha_{ij}^{2}=1$. For more detailed review *see* [1] and [2]*.*

We use the *World Governance Indicators*' dataset covering 214 countries from 1996 to 2019. We use *STATA 17* software to calculate the weights of six dimensions of governance quality, including control of corruption, the rule of law, government effectiveness, rule quality, political stability, and voice and accountability.

Table C1 provides the wights of different dimensions of public institutional quality in each principal component below,

**Table C1. weights of variables in each principal component**

| Variable | PC1 PC2 PC3 PC4 PC5 PC6 |
| --- | --- |
| X1 | 0.3860 0.0796 0.9059 -0.0516 0.1435 -0.0276 |
| X2 | 0.3627 0.8685 -0.2281 0.2269 0.0770 0.0684 |
| X3 | 0.4238 -0.2878 -0.2653 0.0966 0.6314 -0.5091 |
| X4 | 0.4153 -0.3780 -0.0600 0.6536 -0.2052 0.4601 |
| X5 | 0.4335 -0.0519 -0.0920 -0.1933 -0.7214 -0.4931 |
| X6 | 0.4235 -0.1046 -0.2119 -0.6870 0.1112 0.5296 |

Notes: X1 to X6 refers to voice and accountability, political stability and absence of violence, government effectiveness, regulatory quality, the rule of law, and control of corruption, respectively.

The estimated eigenvalues and their shares of the variation of the governance quality are presented in table C2 as,

**Table C2. eigenvalues of the correlation matrix**

| Component | Eigenvalue Difference Proportion Cumulative |
| --- | --- |
| PC1 | 5.08707 4.67747 0.8478 0.8478 |
| PC2 | 0.409606 0.119645 0.0683 0.9161 |
| PC3 | 0.289961 0.169124 0.0483 0.9644 |
| PC4 | 0.120837 0.0728232 0.0201 0.9846 |
| PC5 | 0.0480135 0.0035043 0.0080 0.9926 |
| PC6 | 0.0445092 0 0.0074 1.0000 |

As can be seen, PC1 explains more than 84 percent of the variation of six dimensions of governance quality. However, the second and third principal components define less than 7 and 5 percent of the variation of the governance quality.

[3] introduced a "Scree Plot" to discover statistically significant components. In this approach, also called a scree test, the eigenvalues and principal components are represented as a downward curve in one graph. A point where the slope of the curve flattens out, the "elbow," identifies how many components should be considered. Figure C1 depicts the Scree Plot as follows,

Figure C1. Scree test for determining optimal principal components


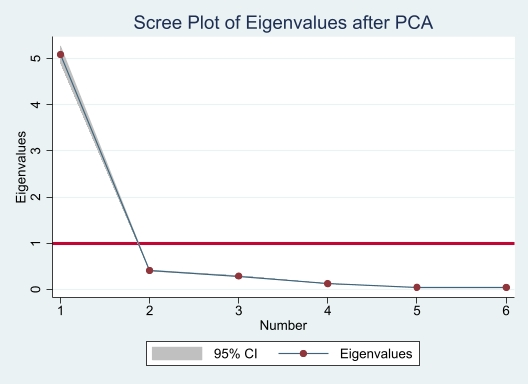


As seen, the Figure displays the eigenvalues on the y-axis along with the number of components on the x-axis. Due to the leveled-off slope of the curve after the second principal component, the first principal component is the most favorable. It is in the same line with the Guttman-Kaiser criterion, taking the number of eigenvalues greater than one as the number of significant components (see [4]).

**References**

**1. Nardo, M., Saisana, M., Saltelli, A., and Tarantola, S. (2005)**, “Tools for composite indicators building,” *European Comission, Ispra*, *15*(1), 19-20.

**2. Vyas, S., and Kumaranayake, L. (2006)**, "Constructing socio-economic status indices: how to use principal components analysis," *Health policy and planning*, *21*(6), 459-468.

**3. Cattell, R. B. (1966)**, "The scree test for the number of factors," *Multivariate behavioral research*, *1*(2), 245-276.

**4. Yeomans, K. A., and Golder, P. A. (1982)**, "The Guttman-Kaiser Criterion as a Predictor of the Number of Common Factors," *Journal of the Royal Statistical Society. Series D (The Statistician)*, *31*(3), 221–229.

**Appendix D: Descriptive statistics of variables**

|  | **Mean** | **Median** | **Maximum** | **Minimum** | **Std. Dev.** |
| --- | --- | --- | --- | --- | --- |
| **Infection per population** | 0.67 | 0.15 | 10.3 | 0.0001 | 1.41 |
| **School closure** | 2.5 | 3 | 3 | 0 | 1.06 |
| **Business closure** | 1.83 | 2 | 3 | 0 | 1.12 |
| **Stay at home requirement** | 1.51 | 2 | 3 | 0 | 1.01 |
| **Contact tracing** | 1.25 | 1 | 2 | 0 | 0.74 |
| **Economic support to households** | 34.08 | 37.5 | 100 | 0 | 30.11 |
| **Test per population** | 125.6 | 37.82 | 1821.66 | 0.001 | 241 |
| **Income inequality** | 40.74 | 41.2 | 62.5 | 25.5 | 8.17 |
| **Health care workers** | 4.57 | 2.88 | 17.28 | 0.35 | 4.28 |
| **Governance quality** | -0.24 | -0.15 | 1 | -1.33 | 0.5 |
| **Tourism arrivals per population** | 0.28 | 0.17 | 1.33 | 0.004 | 0.32 |
| **Average years of adults' education** | 8.06 | 8.2 | 12.3 | 2.7 | 2.62 |
| **Density of population** | 143.63 | 85.11 | 1116.01 | 6.89 | 195.41 |

**Appendix E. the initial estimates with the conventional, scaled, and bootstrapping LASSO methods**

|  | **LASSO** | **Scaled LASSO** | **Bootstrapping LASSO** |
| --- | --- | --- | --- |
| **School closure** | -0.0267 | -0.0440 | -0.0234 |
| **Business closure** | 0 | 0 | 0 |
| **Stay at home requirement** | -0.0493 | -0.0520 | -0.0455 |
| **Contact tracing** | -0.0197 | -0.0456 | -0.0144 |
| **Test per population** | 0.0035 | 0.0036 | 0.0034 |
| **Economic support to households** | 0 | -0.0008 | 0 |
| **Health care workers** | 0 | -0.0158 | 0 |
| **Average years of adults' education** | 0 | 0 | 0 |
| **Income inequality** | 0.0057 | 0.0126 | 0.0040 |
| **Density of population** | 0 | 0 | 0 |
| **Tourism arrivals** | 0.1798 | 0.3223 | 0.1617 |
| **Governance quality** | -0.1506 | -0.2460 | -0.1255 |
| **Africa** | -0.1383 | -0.2262 | -0.1206 |
| **Asia** | 0 | 0 | 0 |
| **Latin America** | 0.1488 | 0.1667 | 0.1502 |
| **Europe** | 0.2525 | 0.3670 | 0.2434 |

**Appendix F: Scatter plots between infection rate and different instruments of government health policy in LMCs**

|  |  |  |
| --- | --- | --- |
| **Corr. Coef. = -0.42** | **Corr. Coef. = -0.32** | **Corr. Coef. = -0.20** |
|  |  |  |
| **Corr. Coef. = -0.08** | **Corr. Coef. = -0.10** | **Corr. Coef. = 0.71** |

Source: Authors’ calculations
